# Vehicular Sensor Network and Data Analytics for a Health and Usage Management System

**DOI:** 10.3390/s20205892

**Published:** 2020-10-17

**Authors:** Kavindu Ranasinghe, Rohan Kapoor, Alessandro Gardi, Roberto Sabatini, Vishwanath Wickramanayake, David Ludovici

**Affiliations:** 1 School of Engineering, RMIT University, Melbourne, VIC 3000, Australia; kavindu.ranasinghe@rmit.edu.au (K.R.); rohan.kapoor@rmit.edu.au (R.K.); alessandro.gardi@rmit.edu.au (A.G.); 2 Land Engineering Agency, Department of Defence, Melbourne, VIC 3006, Australia; vish.wickramanayake@defence.gov.au (V.W.); david.ludovici@defence.gov.au (D.L.)

**Keywords:** sensor networks, health and usage monitoring system (HUMS), vehicle health management, artificial intelligence, machine learning

## Abstract

Automated collection of on-vehicle sensor data allows the development of artificial intelligence (AI) techniques for vehicular systems’ diagnostic and prognostic processes to better assess the state-of-health, predict faults and evaluate residual life of ground vehicle systems. One of the vital subsystems, in terms of safety and mission criticality, is the power train, (comprising the engine, transmission, and final drives), which provides the driving torque required for vehicle acceleration. In this paper, a novel health and usage monitoring system (HUMS) architecture is presented, together with dedicated diagnosis/prognosis algorithms that utilize data gathered from a sensor network embedded in an armoured personnel carrier (APC) vehicle. To model the drivetrain, a virtual dynamometer is introduced, which estimates the engine torque output for successive comparison with the measured torque values taken from the engine control unit. This virtual dynamometer is also used in conjunction with other sensed variables to determine the maximum torque output of the engine, which is considered to be the primary indicator of engine health. Regression analysis is performed to capture the effect of certain variables such as engine hours, oil temperature, and coolant temperature on the degradation of maximum engine torque. Degradations in the final drives system were identified using a comparison of the temperature trends between the left-hand and right-hand final drives. This research lays foundations for the development of real-time diagnosis and prognosis functions for an integrated vehicle health management (IVHM) system suitable for safety critical manned and unmanned vehicle applications.

## 1. Introduction

The recent drive to increase efficiency in vehicle systems has led to an increased interest in developing vehicle health management systems to improve condition-based maintenance (CBM) [1] programs across air, space, and ground platforms. These programs help reduce the cost of maintenance, repair, and overhaul (MRO) of individual and fleet assets. However, from a military standpoint, the key advantages brought forward by CBM are the increased availability and dispatch reliability of vehicle assets and the reduction in any unplanned downtime or accidents related to vehicle faults. The development of health and usage monitoring systems (HUMS) has been the principle enabler in support of CBM. This involves embedding a network of sensors on-board the vehicle that harvest health data across various subsystems and stores it for further processing. This provides an opportunity to leverage the data to develop an intelligent vehicle health management system with the intention of increasing the levels of efficiency and effectiveness of individual assets and vehicle fleets, which can translate into tangible mission, maintenance, and support benefits. The overall benefits expected from an opportune exploitation of HUMS technology are improved availability, safety, and reliability of vehicles and components as well as the minimization of operational, maintenance, and life-cycle costs also in relation to a reduction in the redundancy levels [2,3]. The concept of HUMS was initially introduced by the National Aeronautics Space Administration (NASA) in 1992, as a technology to collect data, diagnose, predict, and mitigate faults and support the operational decisions and post-operational maintenance activities of space vehicles [4]. Current HUMS technologies encompass many vehicle industries such as aircraft, ships, and automobiles, particularly in the defence sector [5,6].

Several studies have been conducted to explore different applications and effective implementation strategies of the HUMS concept to bring forward enhanced maintenance capabilities and techniques, covering many aspects ranging from design and development to verification and operation [7,8]. Advancements in diagnostics and prognostics methods as well as enhanced sensorization capabilities of physical assets has led to this increased interest in health and usage management research, particularly for power train systems [9]. In a study by Kulkarni and Corbetta (2019) [10], a health monitoring framework is developed for the power train of electric unmanned aerial systems (UAS) by integrating a failure mode, effects and criticality analysis (FMECA) based on the qualitative Bayesian approach with a model-based diagnostic and prognostic framework. Le et al. (2017) used machine-learning models to predict the remaining useful (RUL) engine oil in ground-based military vehicles, with data gathered by HUMS on engine RPM, temperature, throttle position, oil temperature, and other key parameters. Other relevant studies in this area include an integrated framework for health assessment and fault classification of the drive train of wind turbines by Zhao et al. (2013) [11] and an online fault diagnosis method for a power train in fuel cell vehicles by Yun et al. (2008) [12]. Within these studies, the importance of the positioning on-board sensors in essential subsystem components is highlighted, particularly those that have a high probability failure rate. A study by Dong et al. (2019) [13] presents the requirements, selection principles, and future development trends of sensors to support the health management of armoured vehicles as well difficulties in sensor installation, taking the vehicle gear box as a case study.

### 1.1. Scope and Structure of the Article

In this paper, data gathered from a sensor network embedded in a ground-based tracked military vehicle is used for the development of a framework of models to assess the health of its power train system. The remaining portion of Section 1 introduces the steps involved in evaluating the state-of-health of vehicle systems from HUMS data as well as the role of artificial intelligence (AI) techniques in diagnosis and prognosis tasks. The sensor network embedded into the military vehicles used for the purposes of this study is described in Section 2. Section 3 presents the methodology of vehicle model development and health indicator identification, while the results and analysis from the modelling is discussed in Section 4, followed by conclusions in Section 5.

### 1.2. HUMS Concept

**Figure 1:**
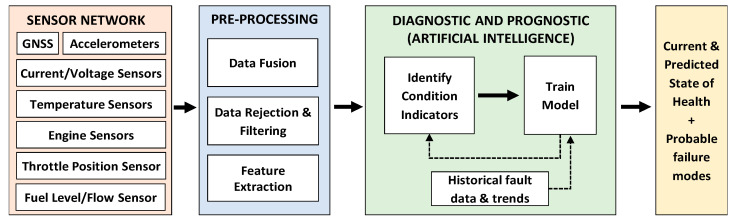
Health and usage monitoring system (HUMS) data workflow diagram.

A basic overview of the data workflow associated to the vehicle usage and health management process using HUMS data is shown in Figure 1.

Firstly, the data are gathered from a number of sensors across the vehicle. These sensors range from conventional embedded sensors to more advanced smart and wireless sensors. Additionally, global navigation satellite system (GNSS) data from L1 frequency observations (1575.42 MHz) are utilized to provide a coherent time and space position information (TSPI) reference. The HUMS onboard CBM-enabled military vehicles, such as an armoured personnel carrier (APC), comprises a sensor network, which captures and stores large amounts of status data at vehicle, system, and component levels [13].

In the pre-processing step, raw measurement data collected from all sensors are filtered, fused, and analysed. Data rejection and filtration are required in this step to remove outliers and noise, to get a realistic picture of normal behaviour. If the data have come from different sources, these will also need to be combined. Instead of feeding sensor data directly into machine-learning models, it is common to extract features from the sensor data. These features capture higher-level information in the sensor data, for example, moving averages or frequency content.

In the next step, the parameters acting as condition indicators for faults are identified and monitored to detect, identify, and characterise faults by studying anomalies and trends. Diagnostic processes allow the rapid determination of specific components/systems that need to be replaced during maintenance and can also contribute to a better understanding on the factors causing any premature failure. Prognostic processes, on the other hand, enable the prediction of the residual life of components/systems and the most likely failure mode by analysing trends in historical observations and implementing model-based estimations [14].

### 1.3. Role of Artificial Intelligence Techniques in Intelligent HUMS

Once the health indicators for all characteristic faults of each critical subsystem of a vehicle have been clearly identified and defined, artificial intelligence techniques can be employed to leverage their ability to identify trends, gain insights from the massive volume of data, and make inferences from them. Machine learning is a subset of AI that refers to techniques/systems designed to take in information within a specific domain and learn from it. These types of reasoning systems have the capability to evaluate and categorize received data, and draw inferences from the data, which could be an insight, decision, or conclusion. A diagnostic system based on machine-learning techniques has the capability to automatically detect the best predictors of system failure, by detecting failure patterns in the training dataset [15]. However, an intelligent vehicle level health management would require a robust reasoning system that could clearly distinguish between the different layers of the vehicle and accommodate data from multiple systems [14].

Since the various subsystems of an APC have unique health indicators that relate to different types of faults, suitable algorithms must be chosen for data processing, feature selection, and extraction. The choice of algorithm would be based on the requirements of the system and the data being processed for insights. Table 1 lists some promising AI approaches that have been grouped according to their characteristics as well as the type of dataset they are most suited for. Group A comprises statistical-reliability-based techniques that assess the health of individual system components solely based on historical rate of failure data of the same component such as the mean time between failures (MTBF), not considering any operating and environmental conditions. These include Weibull analysis and log-normal and Poisson laws [16]. Group B comprises techniques, which can exploit a physics-based model to estimate system dynamics that involve functions of time. These include the Kalman filter [17] and its variants such as the Extended Kalman filter and the unscented Kalman filter [18], as well as particle filters [19]. Group C comprises techniques that rely on physics-based models and are appropriate for data that are not dependent on time. For example, generalized non-linear regression (gradient/Newton-based) techniques [20], particle swarm optimisation (PSO) [21], and decision trees [22] would be included in this category. Algorithms in Group D are appropriate general methodologies for time varying datasets, however they are purely data-driven and depend on a large volume of data to make interpretations. These include techniques such as bi-directional recurrent neural networks (RNN) [23], which excel at analysing sequential data. Group D comprises model agnostic techniques suitable for aggregated analysis such as deep neural networks (DNN) [24], support vector machines (SVM) [25], hidden Markov models [26], and k-nearest neighbour classification [27]. Lastly, Group F include hybrid techniques that combine several model-based and/or data-driven approaches to compensate for the drawbacks in individual approaches, such as adaptive neuro-fuzzy inference systems (ANFIS) [28].

The use of AI techniques would be vital to identify trends in vehicle performance and make inferences of the current and future state of health of safety-critical subsystems [15]. While this enhances the capabilities of CBM by improving the accuracy and reliability of condition assessments and health predictions, the potential of AI extends much further into the field of intelligent integrated vehicle health management (IVHM). Further development of this technology in the future would be an essential factor for the safe and reliable operation of autonomous vehicle applications. In fact, BAE Systems has developed, tested, and demonstrated the capabilities of a fully autonomous APC in an effort to reduce the endangerment of soldiers in future battlefields [29]. Despite the focus on autonomous systems, human operators continue to play a central role in the decision-making process during operations, particularly those involving faulty systems and components. Achieving trusted autonomous capabilities and transitioning from the current “human-in-the-loop” paradigm to a future “human-on-the-loop” operational concept would require a robust IVHM system driven by a combination of model-based and data-driven AI reasoning techniques. This would allow real-time monitoring of the vehicle health conditions during operations, reliable prediction of system faults, and rapid reconfiguration of the available vehicle resources to prevent catastrophic events and to mitigate the effects of single/multiple failures on the overall vehicle performance.

## 2. APC Sensor Network

**Figure 2:**
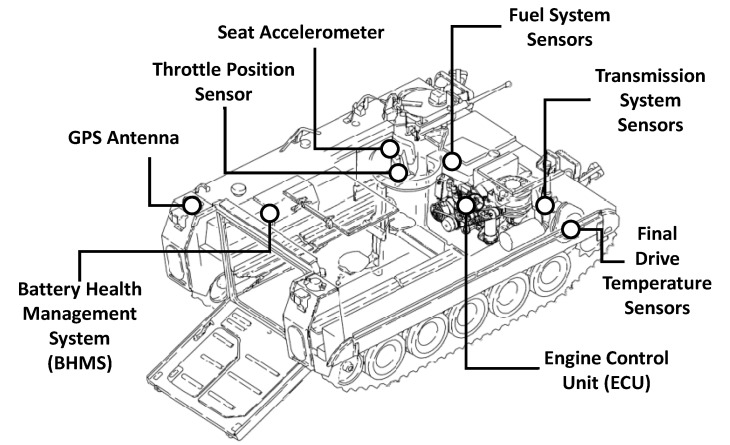
Armoured personnel carrier (APC) fitted with HUMS sensor network.

The data utilized for this study were taken from a fleet of 149 APCs fitted with standard HUMS equipment, including over 50 data channels from an on-board sensor network linked via a controller area network (CANBus) in each APC, as shown in Figure 2. A vehicle data logger is used to capture and store data throughout all mission profiles carried out by each APC. These profiles include both periods of vehicle activity and idle periods during normal operation. At the end of each session, the stored data are converted into a standard format [30] before being compressed and sent to a data historian via an encrypted message queue over Wi-Fi or the commercial 3/4G network. The standard DEF(AUST) 11008 [31] is used to define the requirements of the HUMS as well as the basis for the interface control document, which details the format for the encrypted files and its method of delivery to the data historian.

A selection of the sensor variables obtained from the sensor network that were used in the development of the drive train subsystem models in this study are presented in Table 2. A unique advantage of having a fleet is the availability of aggregate information that can be gathered from each HUMS-enabled APC. Problems can be detected earlier in the life cycle of a vehicle, which can potentially keep the total impact of the problem and the cost of repair relatively low. Furthermore, by studying the faults of similar vehicles operating under similar driving conditions, useful information about faults can be inferred and sent back to specific vehicles to focus performance monitoring on components and systems found to be at risk, based on overall fleet information.

## 3. Methodology

**Figure 3:**
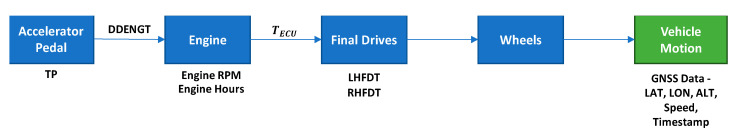
Overview of APC power train.

This section describes the development of physics-based models and identification of health indicators for some of the safety-critical power train subsystems of the APC, namely, the drive train, the engine, and the final drives. Figure 3 provides an overview of the operation of the power train with an indication of the parameters being monitored by the sensor network.

The following sections address the modelling of these subsystems in detail. The models are useful in determining the condition health indicators for the faults associated with their respective subsystem.

### 3.1. Virtual Dynamometer

The purpose of a dynamometer is to measure the force, torque, or power generated by the engine of a vehicle. These measurements can then be utilised as a basis for detailed investigations into the performance and health characteristics of the engine. Traditionally, dynamometers are devices connected to the engine in a test environment that simulate road loading and gather the required data. However, taking advantage of the suite of sensors available on the HUMS-enabled APC, a virtual dynamometer can be constructed by analysing the relevant parameters during normal operation of the vehicle. The virtual dynamometer acts as a physics-based observer or virtual sensor, which fuses data from different sensors across the sensor network and provides an alternate source of engine output torque measurement. This measurement proves to be valuable when developing diagnostic and prognostic algorithms for the drivetrain as well as the model for engine degradation.

**Figure 4:**
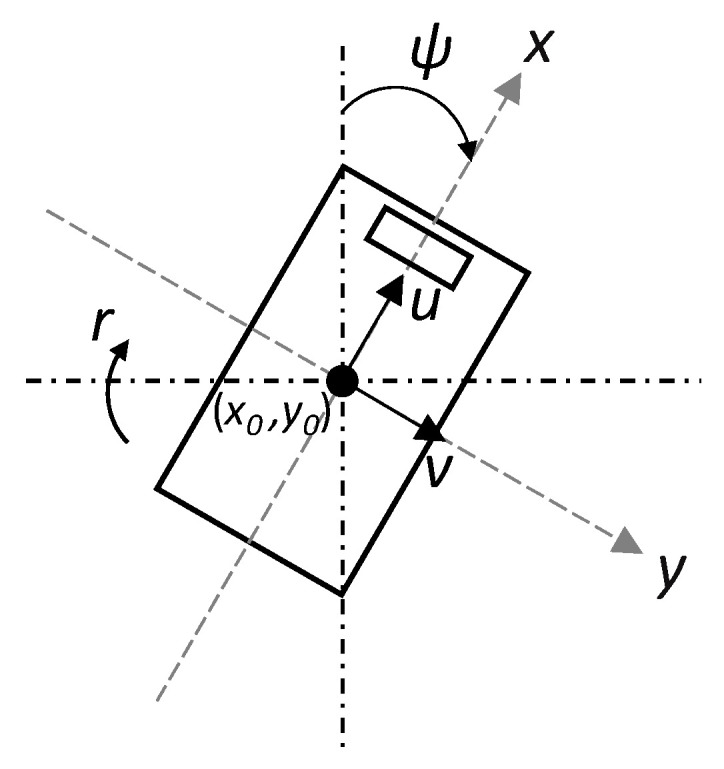
Degrees of freedom of the APC.

The first step in the development of the virtual dynamometer is to establish a mathematical model to analyse the motion of the APC. A standard 6 degree of freedom vehicle dynamics model comprises differential equations describing 3 motions of translation (longitudinal, lateral, and vertical) and 3 motions of rotation (pitch, roll, and yaw) [32]. It is also important to differentiate between the body axes system and the road (inertial) axes as shown in Figure 4.

In consideration of a virtual dynamometer, it can be assumed that for ground vehicle applications, motion along the vertical *z*-axis as well as pitch and roll motion are negligible. Additionally, under the assumption of no slide-slip, the velocity in the lateral *y*-axis will always be zero. This analysis was simplified by neglecting the resistance encountered during turning manoeuvres. This is justified with the assumption that all vehicles in the fleet are operated similarly and measurement and trending over extended periods of time will encompass similar levels of turning for all vehicles. Therefore, the only degrees of freedom to consider are motion along the longitudinal x-axis and yaw motion. The variables describing position, orientation, and velocity are:

- $x_{0}$, $y_{0}$—position of vehicle centre of mass in the road (inertial) axes.
- ψ—yaw angle (between longitudinal axis and north).
  u, v—velocity in the longitudinal and lateral body axes, respectively.
- r—yaw rate.

The kinematic equations derived from the 6 DOF model are:

(1) $${\dot{x}}_{0}=ucos\psi -vsin\psi$$

(2) $${\dot{y}}_{0}=usin\psi +vcos\psi$$

(3) $$\dot{\psi }\;=r$$

However, in this case v will always be equal to zero. Concurrently, the force and moment equations that describe the longitudinal and yaw motion are:

(4) $$ma_{x}=F_{right}+F_{left}-F_{G,x}-\sum^{\;}F_{R}$$

(5) $$J_{vehicle}\ddot{\psi }\;=b\cdot \left(F_{right}-F_{left}\;\right)$$

where:

- m, $J_{vehicle}$—mass and inertia of the vehicle, respectively.
- $a_{x}$—longitudinal acceleration (obtained from GNSS data).
- $F_{right}$, $F_{left}$—tractive effort produced by right and left tracks, respectively.
- $F_{G,x}$, $F_{R,x}\;$—gravitational and resistance forces (comprised of aerodynamic resistance $R_{A}$ and rolling resistance $R_{R}$).
- b—perpendicular distance between tracks and centre of moment

For the purposes of the virtual dynamometer, it is convenient to adapt these equations for solely movement in a straight line, keeping only variables associated with the longitudinal axis. It is also assumed that the vehicle is symmetric, and the torque is evenly distributed to both the left and right track.

**Figure 5:**
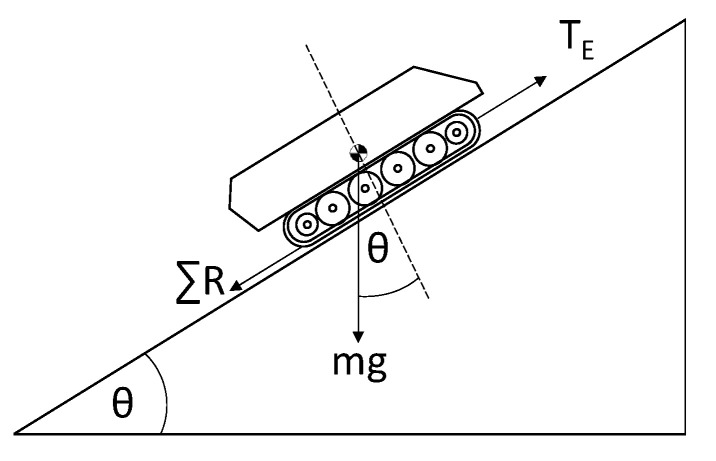
Force diagram of APC during normal operation.

(6) Fright=Fleft=TE2

where $T_{E}$ represents the total tractive effort produced by both tracks of the APC. A force diagram to represent the motion of the APC in a straight line is shown in Figure 5.

Equation (4) can then be re-written as:

(7) $$T_{E}=ma_{x}+mgsin\theta +\sum^{\;}F_{R}$$

(8) $$\sum^{\;}F_{R}=\;R_{A}+R_{R}\;$$

where m is the mass, g is the gravitational acceleration constant, and θ is the angle of elevation.

**Figure 6:**
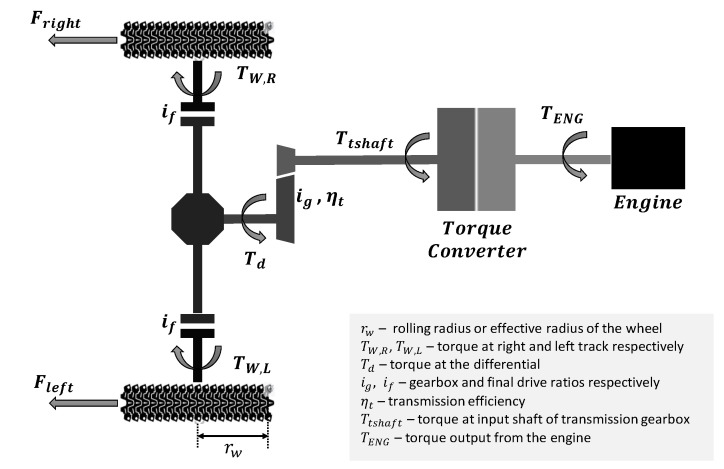
Simplified APC drive train schematic.

To relate the tractive effort produced at the tracks to the torque output by the engine, it is necessary to understand how the torque is transferred by the drive train of the vehicle. Figure 6 shows a simplified drive train schematic to help illustrate the different torque relations within it.

The torque produced by the left and right track can be related to their respective tractive efforts via the following equations:

(9) $$T_{W,R}=F_{right}\cdot r_{w},\;T_{W,L}=F_{left}\cdot r_{w}$$

From Equation (6), for movement in a straight line, $F_{right}$ would be equal to $F_{left}$, therefore the corresponding torques would also be the same. The left and right wheel torque are related to the torque at the differential $T_{d}$ by the following equation:

(10) $$T_{d}=\left(T_{W,R}+T_{W,L}\right)/i_{f}=\left(T_{E}\cdot r_{w}\right)/i_{f}$$

$T_{d}$ can be related to the torque at the input shaft of the transmission gearbox $T_{tshaft}$ through the following equation:

(11) Ttshaft=Tdigηt

Between the engine and the transmission gear box is the torque converter (TC) made up of a pump (driving member connected to engine) and a turbine (driven member connected to the transmission). The torque relation between the transmission and the turbine is given by:

(12) $$T_{tshaft}=T_{turbine}-{\dot{\omega }}_{t}I_{t}$$

where $T_{turbine}$ is the torque at the turbine and ${\dot{\omega }}_{t}$ and $I_{t}$ are angular acceleration and inertia of the turbine of the torque converter, respectively. In the torque converter, torque is transferred to the pump by the turbine can be calculated by the following equation:

(13) $$T_{pump}=T_{turbine}/TR\left(SR\right)$$

where TR is the torque ratio of the torque converter. TR itself is a function of the rotational speed ratio (SR) between the turbine and the pump. The relationship between TR and SR can be obtained from a steady-state look-up table of parameters of the torque converter specified by the manufacturer. Finally, the torque output from the engine $T_{ENG}$ can be related to the pump torque through the following equation:

(14) $$T_{ENG}=T_{pump}+{\dot{\omega }}_{p}I_{p}$$

where ${\dot{\omega }}_{p}$ and $I_{p}$ are angular acceleration and inertia of the pump of the torque converter, respectively.

The following subsections detail the methods by which the variables involved in the above equations are obtained to perform the calculation of the tractive effort $T_{E}$ in Equation (7).

#### 3.1.1. Resistances

The aerodynamic resistance $R_{A}$ can be modelled by the following equation:

(15) $$R_{A}=\frac{1}{2}\rho v^{2}SC_{D}$$

where ρ is the atmospheric density, v is the velocity, S is the projected frontal area of the vehicle, and $C_{D}$ is the coefficient of aerodynamic drag.

The rolling resistance $R_{R}$ can be simply modelled as a function of the normal reaction force applied by the vehicle and the coefficient of rolling resistance:

(16) $$R_{R}=\;\varphi mg\cos\theta$$

where the value of coefficient of rolling resistance φ depends primarily on the type of terrain that the vehicle is exposed to. Utilising the idea of terrain classification based on average speed over 100 m segments, 3 classes of terrain and their respective empirical values of φ can be identified, as detailed in Table 3.

#### 3.1.2. Gravitational Force Component

To account for the effect of the weight of the vehicle in the calculation, it is important to determine the angle of elevation θ. Using the GNSS data for latitude, longitude, and altitude and the Haversine calculation [33] of the distance between two consecutive points, Δd, the following equation can be implemented to obtain *θ*:

(17) $$\theta =\arctan\left(\frac{\Delta z}{\Delta d}\right)$$

where Δz is the change in altitude between two consecutive points.

#### 3.1.3. Gear Setting Identification

**Figure 7:**
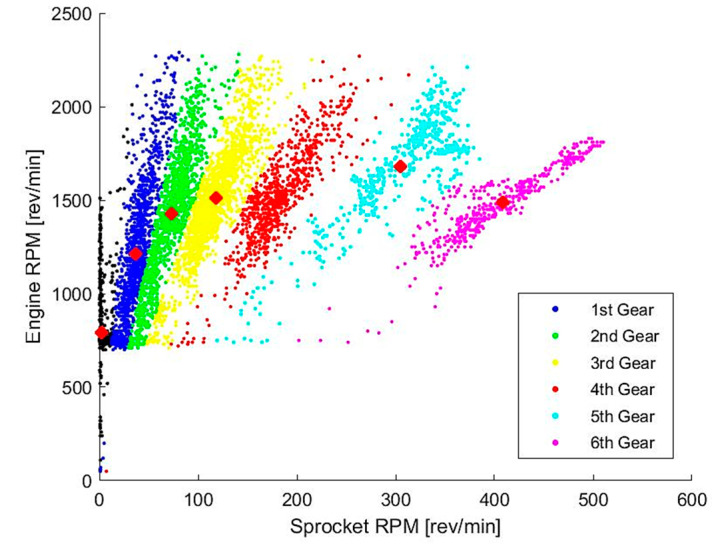
Clustered engine revolutions per minute (RPM) vs. sprocket RPM (single operating session).

While $i_{f}$ is a constant for the final drives of the APC, $i_{g}$ and $\eta_{t}$ vary according to the gear setting. Therefore, it is necessary to develop a method of determining the instantaneous gear setting of the vehicle during operation. This can be done by comparing the revolutions per minute (RPM) between the output shaft of the engine and wheel sprocket, as shown in Figure 7. The engine RPM is a measured parameter from the on-board sensors, whereas the sprocket RPM can be calculated from its relation to the speed, as shown in the following equation:

(18) $$SP_{rpm}=SP_{rmil}\cdot v$$

where v is the velocity and $SP_{rmil}$ is the number of sprocket revolutions per mile, which is a known constant for the APC.

From Figure 7, it is evident that data points with a similar gear ratio are clustered and divided into 6 respective gear settings. This provided a good opportunity to make use of a clustering algorithm, which can assign data points to different groups based on their similarity. In this instance, a fuzzy c-means clustering (FCM) algorithm [34] was utilized to group the data points into 7 clusters (6 gears settings and 1 outlier group). The algorithm computes the coordinates of the centroid of each cluster and assigns coefficients to each data point (membership grades), which indicates the degree to which data points belong to each cluster.

Once the required parameters have been obtained, $T_{ENG}$ can be calculated and its variation compared to that of the measured engine torque from the ECU ($T_{ECU}$). This comparison can be the basis of the general health indicator of the drivetrain. 

### 3.2. Maximum Torque Degradation of Engine

Through wear and tear, engines are subjected to degradation, leading to a loss of maximum torque output over time. There are several factors that could cause degradation in engine performance, such as restrictions in the air intake, clogged fuel injectors, loss of compression due to cylinder wall wear, coking residue on bearings, bearing defects, bypass gas leakage, exhaust valve failures, and cooling pump failures [35,36,37].

**Figure 8:**
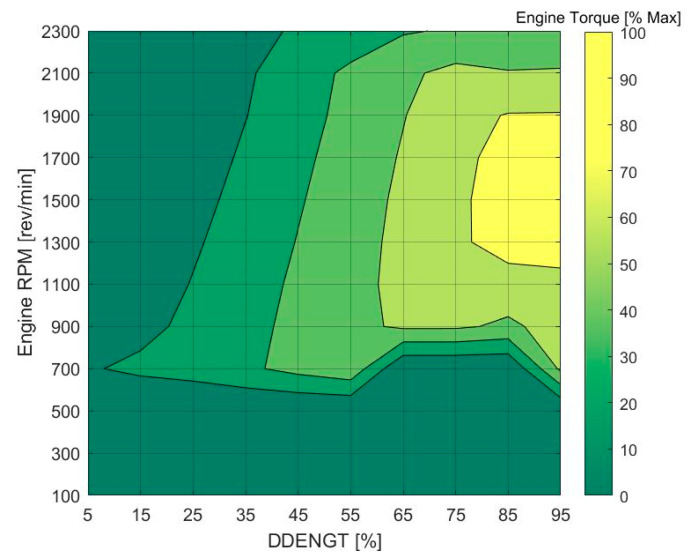
Torque map of APC engine.

The virtual dynamometer proves to be a useful tool in obtaining the maximum torque output by the engine in a given operation session. Considering the torque map given in Figure 8, it can be inferred that maximum torque is achieved in the region between engine speed of 1200 RPM to 1900 RPM as well as driver demand engine torque ($DD_{ENGT}$) greater than 80% (representative of the throttle/accelerator pedal position). After applying these two filters to the virtual dynamometer torque data, the maximum torque for a given operating session of a given APC can be determined.

Figure 8 shows the graph of maximum torque over the operating period of an APC, measured in engine hours. Engine hours was chosen as an accurate representation of the usage of the APC rather than the physical time of service. 

**Figure 9:**
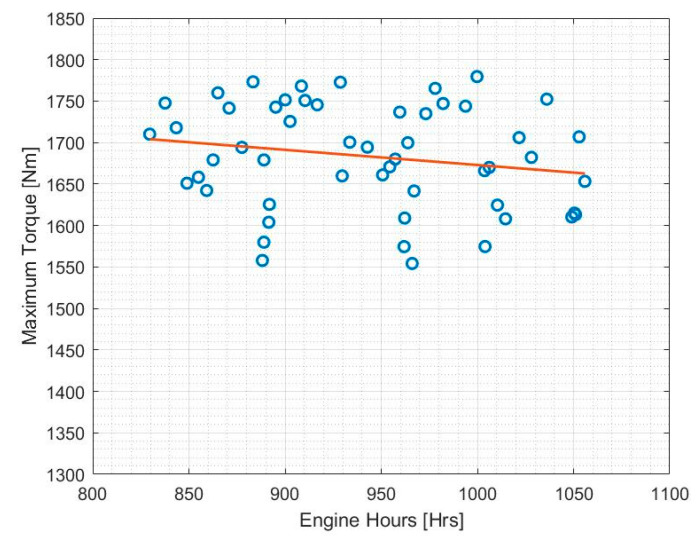
Maximum torque variation with engine hours.

A simple least-squares fit line was used to analyse the variation of maximum torque over time in Figure 9. It is evident that this particular APC engine operated at a fairly even performance level with a gradual decrease in maximum torque over the operating period under observation.

This method can also be used to determine how operating conditions effect the rate of degradation of engine torque. Variables that are currently measured that could affect the degradation of the maximum torque output of the engine include:

- % time with throttle > 70%—extended periods of time with high driver demand engine torque in a single operation puts more strain on the engine;
- Engine oil temperature—lower engine oil temperature leads to higher viscosity and the engine running less efficiently;
- Engine coolant temperature—inefficient cooling will affect engine performance;
- Ambient temperature—internal combustion engines develop more power in cold conditions (along with high barometric pressure) when the charge air density is high.

The trend can be projected further to predict when the maximum torque variation would exceed predefined thresholds at which alerts can be triggered to prompt engine servicing or replacement. In order to make this prediction as well as take into account the variables that affect the maximum torque output of the engine, a regression-learning technique was used. Due to its ability to analyse multivariate data, regression analysis is particularly useful in situations where the health indicator (the response variable) demonstrates causal relationships between it and many other independent variables of a given system (predictor variables) [38]. For an accurate regression analysis, it is important to be able to justify that these relationships have a considerable effect on the health indicator of the system. This technique can be augmented with the use of principal component analysis (PCA), which is a dimensionality-reduction technique that identifies the most significant trends in a given dataset based on their variance [39].

Different regression model types were trialled during the training process, including linear regression models, support vector machines (SVM), Gaussian process regression models, and ensembles of decision trees. Criteria used to assess the quality of predictions of different model types were the root mean squared error (RMSE), r-squared value, and training time. A linear SVM model was selected for the regression model, as it captured the steady degradation of maximum torque with a suitable degree of prediction performance while avoiding overfitting. SVMs are learning machines implementing the structural risk minimization inductive principle to obtain good generalization on a limited number of learning patterns [40]. SVMs were developed to solve traditional classification problems, as they work by separating the data using hyperplanes, however they can also be extended to the domain of regression problems [41].

### 3.3. Degradation of Final Drives

The final drives of the APC aid in the transferring of torque from the output shaft of the engine to the sprocket shafts that drive the front wheels of the left- and right-hand tracks, respectively. As with any mechanical system, a considerable amount of heat is generated during normal operation. However, common faults pertaining to the final drive lead to excessive temperatures, which can be detected by HUMS sensors. These faults include low gear oil level, wear and tear of the bearing, and defective gears.

**Figure 10:**
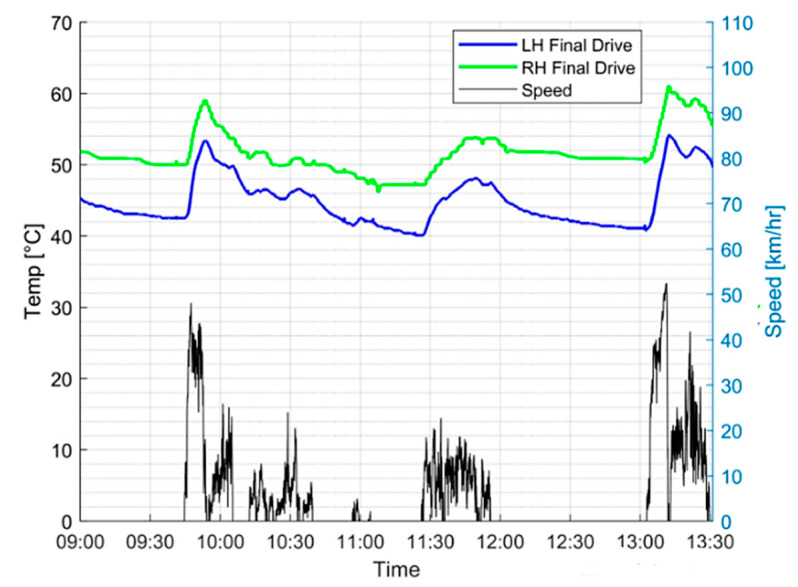
Variation of final drive temperature (vehicle speed shown as reference).

Figure 10 shows the variation of the two final drive temperatures during an operating session of a particular APC. The speed of the vehicle is also plotted for reference to the active periods of the vehicle.

From initial observation, it is evident that a constant discrepancy between the left-hand (LH) and right-hand (RH) final drive temperatures is present. This can be attributed to the closer proximity of the right-hand final drive to the engine, which is inherent in the design of the APCs used in this study. There are also other background heat sources that affects the rise and fall of the final drive temperature such as ambient air temperature and the heat generated by brakes. Therefore, it is difficult to trend the individual final drive temperature without considering these background heat sources.

It was found that monitoring correlation coefficient between the two final drives would help remove the background heat effect from the health analysis of the final drives. This would involve monitoring the correlation variation of the left- and right-hand final drive temperatures during vehicle operation, with the assumption that a fault in either drive would correspond to a discrepancy in the relative variations of these two variables. From Figure 10, it is evident that there is a strong correlation between the two graphs, which is indicative of a healthy final drive system. The Pearson correlation coefficient (CC) formula can be used to quantify the extent of the correlation:

(19) $$CC\left(LH,RH\right)=\;\frac{1}{N-1}\overset{N}{\underset{i=1}{\sum }}\left(\frac{\bar{LH_{i}-\mu_{LH}}}{\sigma_{LH}}\right)\left(\frac{RH_{i}-\mu_{RH}}{\sigma_{RH}}\right)$$

where N is the number of data points, μ is the mean, and σ is the standard deviation.

## 4. Results, Data Analysis, and Discussion

### 4.1. Virtual Dynamometer

**Figure 11:**
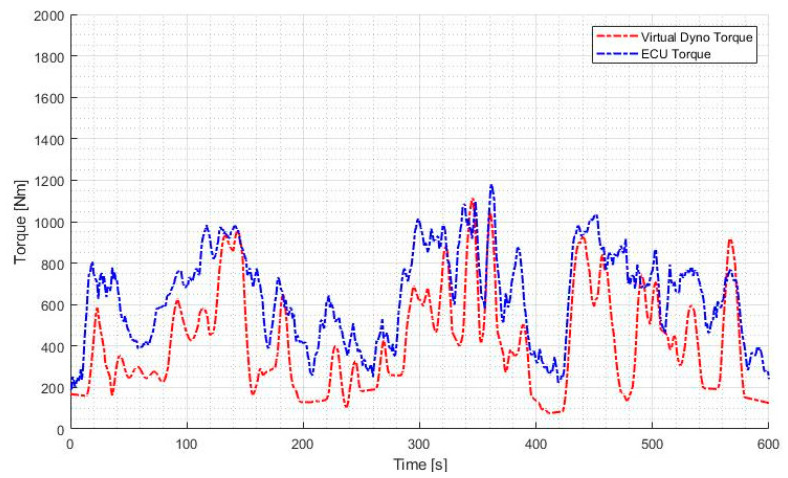
Comparison of virtual dynamometer torque and ECU torque.

**Figure 12:**
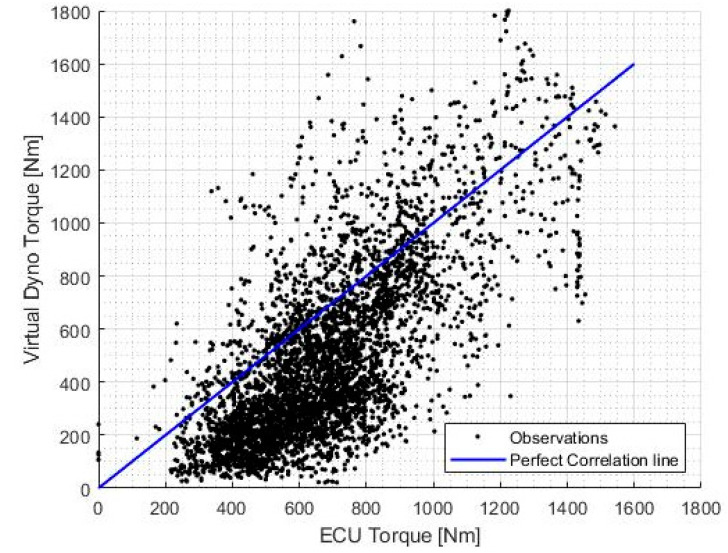
Plot of correlation between torque values.

The comparison between the calculated torque outputs produced from the engine ($T_{ENG}$) and the engine torque from the engine ECU ($T_{ECU}$) for a selected window of operation is shown in Figure 11, as well as the corresponding correlation plot in Figure 12.

The correlation coefficient between the variation of $T_{ENG}$ and $T_{ECU}$ in Figure 11 was found to be 0.7515, which is indicative of the goodness of the prediction of torque by the virtual dynamometer model.

An important characteristic to note is that the calculated torque output of the engine (Equation (14)) is, in general, lower than the value determined by the ECU. This disparity can be attributed to the losses in torque through the drivetrain of the vehicle. To quantify this disparity, the root mean squared difference (RMSD) between the two graphs can be calculated.

(20) $$\mathrm{RMSD}=\;\sqrt{\frac{1}{n}\overset{n}{\underset{i=1}{\sum }}{\left(T_{ECU,i}-T_{ENG,i}\right)}^{2}}$$

A certain amount of disparity between the measured and calculated torque output is expected due to the losses in mechanical systems. However, degradation of the drivetrain would correspond to an increase in this disparity, therefore the average RMSD over a normal operating session can be taken as an indicator of the state of health of the drivetrain.

The concept of using multiple sensor data fusion along with the vehicle dynamics model to construct a virtual sensor proves to be a powerful tool in the development of diagnostic and prognostic algorithms for the power train. The value of it is brought forward by the alternative source of measurement of a given parameter of interest, which can then be compared to sensor reading to generate residual values that can be used in health estimation and fault diagnosis. Additionally, this methodology can be transferred to other vehicle platforms, with appropriate modifications to the physics model due to the variation in power train system architecture as well as the different vehicle dynamics model. 

### 4.2. Maximum Torque Degradation of Engine

**Figure 13:**
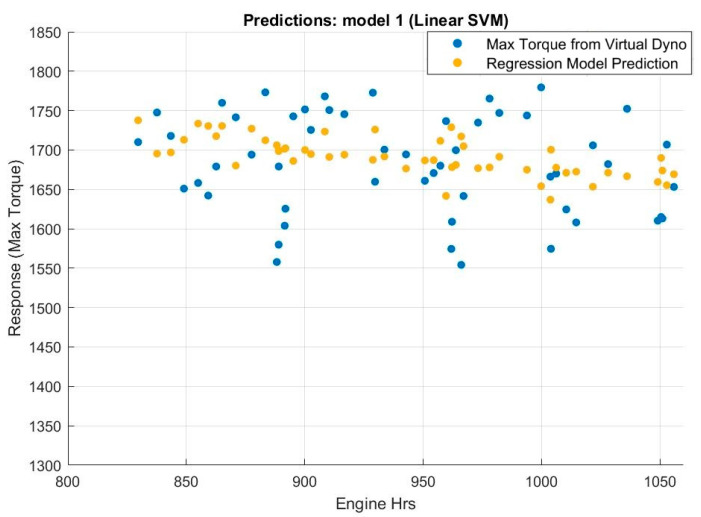
Max torque from virtual dyno (blue) and predicted values (yellow) using linear support vector machines (SVM).

The prediction values of the response variable of the trained regression model based on data taken from a particular APC are plotted in Figure 13 against the number of engine operational hours.

It is evident that there is a clear declining trend in maximum torque with increased usage of the APC. This method of analysing maximum torque degradation takes multiple variables into account, which accounts for the lower degree of scattering of the values predicted by the regression model.

**Figure 14:**
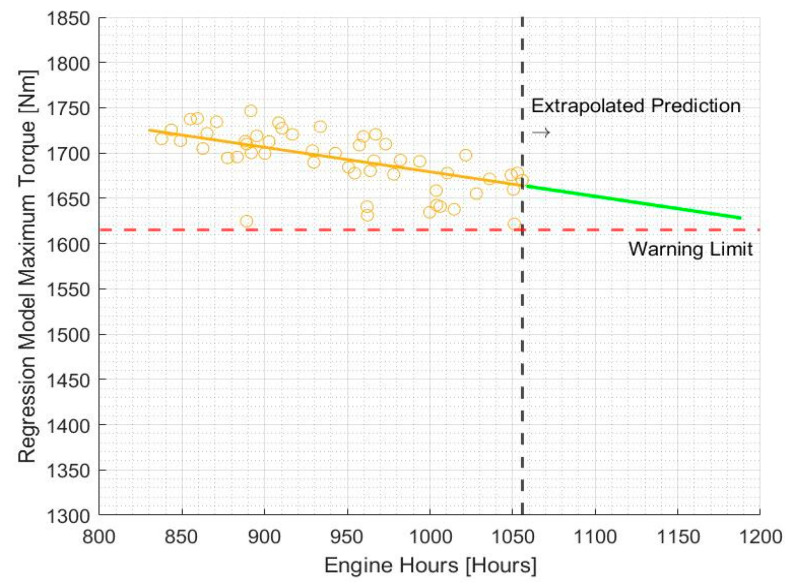
Maximum torque trend predicted by regression model based on linear SVM.

Once the regression model is trained, it can be exported and then extrapolated to make predictions of the behaviour of the maximum torque based on a set of values of the predictor variables. These variables can be adjusted based on several different hypothetical scenarios, for example, if the APC is forecasted to undergo a highly active period of usage in a cold climate in the near future, low values of ambient temperature would be chosen. Such a prediction for continuous normal operation of the APC is made in Figure 14.

In order to inform the maintainer about a health degradation of the engine based on the variation of maximum torque, caution and warning limits need to be developed. The ASTM D7720-11 method [42] based on the fleet cumulative distribution was used. Based on this method, a threshold of 1615 Nm maximum torque output was set as a warning threshold for triggering of an alert for required maintenance. This provides vital information for the operational and maintenance crew of the APC to plan their activities.

### 4.3. Degradation of Final Drives

**Figure 15:**
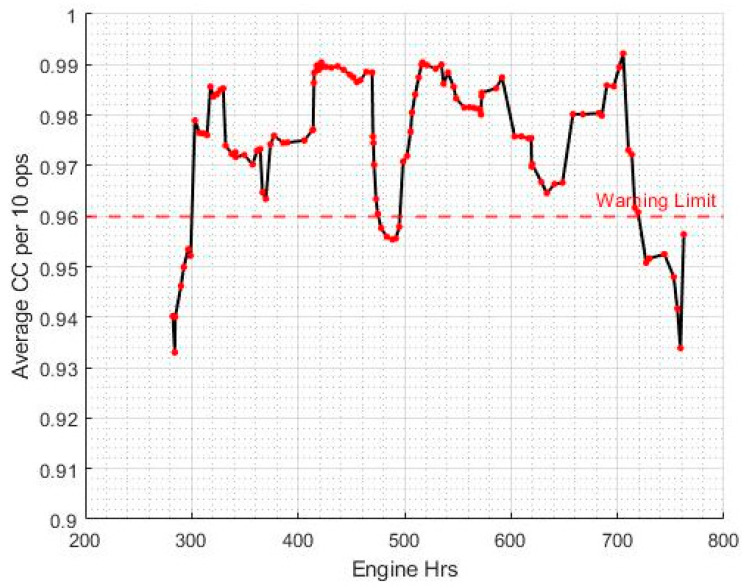
Variation of average CC per 10 operations over time.

Figure 15 shows a moving average of the calculated CC, with a window of 10 operating sessions, for a particular APC known to formerly have had issues with its final drives.

Once again, the ASTM D7720-11 method was used to develop warning limits for this parameter based on fleet cumulative distribution. As evident from Figure 15, the vehicle displays several occasions in its history where the temperature variations of the left-hand and right-hand final drives exhibit a poor correlation coefficient over several consecutive operating sessions. Furthermore, these instances correspond to the times where the APC was suspected to have sustained damage to its final drives, most likely due to gear teeth wear. However, the problem was not picked up on until a technical inspection was carried out months later. This further reinforces the idea of using the correlation coefficient of the two temperature variations as a health indicator for the final drives.

## 5. Conclusions

This study establishes a framework of models and algorithms that utilize HUMS data to provide outputs that accurately portray the current and future state of health of mission and safety-critical power train subsystems of an APC. The framework includes:

- a virtual dynamometer, which acts as a model-based virtual sensor that fuses multiple sensor measurements to calculate the output torque of the engine based on the motion of the vehicle.
- an engine performance degradation model based on regression analysis of the maximum torque output over time.
- a health assessment of the final drives based on the difference between temperature variation of the left- and right-hand final drives.

The outputs of these models were analysed to detect anomalies and predict faults of their respective subsystem by examining trends of deviation from expected normal operating conditions. Although a ground-based application was chosen for this study, the implementation of this methodology to make fault predictions in vehicle health management systems has a strong potential to enhance safety, reliability, and efficiency across many different applications and is a major part of the authors’ ongoing research in cyber-physical and autonomous systems for aerospace and defence applications. In addition to fault predictions, these techniques have the potential to enhance the overall design of mission-essential and safety-critical vehicle systems as well as allowing a real-time dynamic reconfiguration of the available vehicle resources, thereby supporting trusted autonomous operations of future ground, maritime, and aerospace vehicles. 

Future research will focus on the development of digital representations or ‘digital twins’ of vehicle systems which take real-time measurements from sensors as inputs and produce predications or estimations of the system responses to various inputs and external conditions. The introduction of these digital twins has a significant potential to enhance the design of future IVHM systems, as the health assessment and predictions obtained from high-fidelity and real-time simulations aid in early detection of faults, prevention of downtime and planning of future missions or activities. Furthermore, the models will be augmented with both physics-based and data-driven AI techniques to capitalize on the complimentary advantages of these two approaches and to maximise the timeliness and reliability of diagnosis and prognosis information for safety-critical IVHM applications.

## Figures and Tables

**Table 1:** Potential artificial intelligence (AI) approaches for HUMS data.

	Time Trends	Aggregated Analysis
Reliability-based	Group A	-
Physics-model-based	Group B	Group C
Data-driven	Group D	Group E
Hybrid	Group F	

**Table 2:** List of sensor variables.

Subsystem	Sensor Variable	Range/Units	Measurement
Accelerator Pedal	Throttle Position (TP)	0 to 100%	Ratio of actual position of accelerator pedal to maximum position
Engine	Driver demand Engine Torque (DDENGT)	0 to 100%	Instantaneous engine torque demanded by driver. Calculated by engine control unit (ECU)
	Actual Percent Engine Torque ($T_{ECU}$)	0 to 100%	Output torque of engine calculated by ECU
	Engine RPM	0 to 3000 rev/m	Operating speed of engine calculated by ECU
	Total Engine Hours (ECU + HUMS)	N/A h	Aggregate vehicle engine hours incremented when the condition (engine RPM >= 200 for 0.1 s) is satisfied
Final Drives	Left-Hand Final Drive Temperature (LHFDT)	−10 to 150 °C	Temperature of LH final drive measured by k-type stick on thermocouples
	Right-Hand Final Drive Temperature (RHFDT)	−10 to 150 °C	Temperature of RH final drive measured by k-type stick on thermocouples
Vehicle (GNSS)	GNSS Altitude	0 to 8000 m	Height above sea level
	GNSS Latitude	−90 to 90 degrees	Latitude co-ordinate of vehicle
	GNSS Longitude	−180 to 180 degrees	Longitudinal co-ordinate of vehicle
	GNSS Speed	km/h	Vehicle speed derived from GNSS data
	GNSS Time	N/A	Timestamp determined by GNSS
Vehicle (Totals)	Vehicle Distance	N/A km	Total distance travelled (generated from GNSS data)
	Vehicle Hours	N/A h	Aggregate vehicle hours incremented when the condition (alternator voltage output >= 5 volts for 1 s) is satisfied

**Table 3:** Terrain classification.

Terrain Class	Average Speed [km/h]	Coefficient of Rolling Resistance φ
First Class	v > 58.1	0.024
Second Class	30.9 <v < 58.1	0.08
Cross Country	v < 30.9	0.17
